# Nordic Multimorbidity Index, Charlson, Elixhauser and count-based comorbidity indices for mortality risk: a comparative nationwide cohort study using national health registers in Sweden

**DOI:** 10.1136/bmjopen-2025-114635

**Published:** 2026-06-22

**Authors:** Björn Zethelius, Mats Talbäck, Rickard Ljung

**Affiliations:** 1 Use and Information Division, Swedish Medical Products Agency, Uppsala, Sweden; 2 Department of Public Health, Clinical Geriatrics, Uppsala University, Uppsala, Sweden; 3 Institute of Environmental Medicine, Karolinska Institutet, Stockholm, Sweden

**Keywords:** Mortality, STATISTICS & RESEARCH METHODS, EPIDEMIOLOGY, INTERNAL MEDICINE, Prospective studies, REGISTRIES

## Abstract

**Abstract:**

**Objectives:**

The aim of the present study was to compare Area Under the Receiver Operating Characteristic curves (AUROCs) of age-and-sex alone, and in addition respectively, the Nordic Multimorbidity Index (NMI), Charlson Comorbidity Index (CCI) and Elixhauser Comorbidity Index (ECI), and explore simple count-based measures on in-patient care and filled drug prescriptions, with the outcome mortality using Swedish health registries.

**Study design and setting:**

Study population: All persons born in 1975 or earlier who were continuously resident in Sweden between 1 January 2010 and 31 December 2014 (n=5 010 261) with a follow-up for all-cause mortality from 1 January 2015 to 31 December 2019.

**Methods:**

AUROCs were calculated with 1- to 5-year-look-back for age-and-sex, each index and explored measures for 1- to 5-year mortality.

**Results:**

AUROC was for age-and-sex alone for 5-year mortality 0.8731. Age-and-sex alone outperformed, respectively: NMI, difference in AUROC, 0.0695 (95% CI 0.0687 to 0.0704); CCI, 0.1509 (95% CI 0.1500 to 0.1518) and ECI 0.1631 (95% CI 0.1622 to 0.1640). AUROC for NMI when added to age-and-sex, 0.9072 was marginally higher than age-and-sex alone. AUROC for explored measures as numbers of hospitalisations, days hospitalised, or numbers of filled drug prescriptions were around 0.75–0.80 and when added to age-and-sex around 0.90.

**Conclusion:**

Age-and-sex alone showed higher AUROC than any other measure alone and NMI provides modest additional discrimination, with CCI and ECI both less helpful, that is, informative. Explored measures; days hospitalised or numbers of filled prescriptions rendered AUROC of somewhat lower magnitude when added to age-and-sex. Thus, the latter could be considered for use in addition to age-and-sex when full background data are lacking.

**STRENGTHS AND LIMITATIONS OF THIS STUDY**

- Major strengths were the use of population-based nationwide health registers with compulsory registration both in terms of cohort information and on diagnoses and filled prescriptions, that is, high validity.
- It includes 5 million persons aged 40 and above with 5 years of historic data and complete information on mortality over the 5 years follow-up period.
- It includes analyses on three different age groups, with increasing comorbidity burden at higher ages.
- It includes in- and out-patient diagnoses from specialised care and filled prescriptions from all physicians, regardless of workplace, and thus has a lower threshold for defining comorbidity compared with hospitalisation data only.
- A limitation is the lack of diagnoses from primary care; however, filled prescriptions from primary care were included.

## Introduction

 Comorbidity, the co-existence of long-term medical conditions is common and rises with advancing age. In non-randomised studies confounding from comorbidity can be complex and is essential to adjust for when analysing exposure to health outcome relations. In epidemiological studies using national health registries, based on routinely collected healthcare data often provide detailed information on filled prescriptions and diagnoses, whereas information on disease severity, lifestyle factors, general physical condition and general health of individuals is often limited. The Nordic countries have similar government-funded healthcare systems with universal population coverage, and mandatory reporting to nationwide health registries covering the entire population providing a unique setting for epidemiologic research.

In epidemiological studies, adjustment for age and sex is routine. Further, aggregations of diagnoses can be formed into a single comorbidity index variable. A commonly used index for comorbidity is the Charlson Comorbidity Index (CCI) from 1987 which was developed to predict 1-year mortality in hospitalised patients.^1^ It is often used as a proxy for comorbidity burden and as a covariate in regression models. Another example is the Elixhauser Comorbidity Index (ECI) from 1998 which was developed to predict in-hospital mortality and length of hospital stay.^2^ Both indices were derived from in-patient population data in the USA. A Nordic Multimorbidity Index (NMI) was developed based on Danish hospital discharge diagnoses and filled drug prescriptions to predict 5-year mortality in a population-based cohort, constituting a random 20% sample of all Danish individuals aged ≥40 years.^3^ The authors have called for validation in other patient populations and other Nordic countries.

The aim of the present study was, when taking age and sex into account, to compare performance of the NMI to the CCI and the ECI using Swedish register data and describe the impact of different lengths of the look-back period and of follow-up for mortality, that is, to elaborate on one to 5 years of historical data for 1–5 years of mortality prediction to illuminate amount of historical data needed. Further, to explore simple count-based measures based on in-patient care and filled prescriptions, respectively, that is, numbers of diagnoses, numbers of hospitalisations, numbers of days of in-patient care and numbers of filled prescriptions when added to age and sex to explore if such measures may be as useful as the more elaborate indices.

## Materials and methods

### Study population

The study population consisted of all persons born in or before 1975 who were continuously resident in Sweden between 1 January 2010 and 31 December 2014 to allow for full 5-year look back coverage of data from 2010 to 2014. A total of 5 631 018 individuals had lived in Sweden at some point between 2010 and 2014, of these 444 167 (7.9%) died and 176 590 (3,1%) migrated to or from Sweden during this period and were thus not included.

The study population was followed for all-cause mortality between 1 January 2015 and 31 December 2019. We hesitate to consider follow-up to more recent years than 2019 due to the COVID-19 pandemic that spread across the globe early in 2020 and was declared over by the World Health Organisation in May 2023. It had consequences to all parts of life, and we cannot exclude that increased mortality during the pandemic may affect the relation between index values and mortality in analyses.

### Patient and public involvement

Reporting by care givers to national health registers in Sweden is compulsory by law and patients cannot deny being registered. However, there was no involvement of patients or members of the public in the design, data collection, conduct or reporting of this study. As this study was performed at a governmental agency the report is freely available to the public and it may be referred to in Swedish public media or elsewhere.

### Register sources

The following registries, included in the COvid-19 VACcination register SAFEty study in Sweden (CoVacSafe-SE) platform,^4^ were used: Swedish National Prescribed Drug Register (PDR),^5^ Swedish National Patient Register (NPR)^6 7^ and Swedish National Cause of Death Register (CDR)^8^ held by the National Board of Health and Welfare and the Swedish Total Population Register^9^ and the Swedish Longitudinal Integrated Database for Health Insurance and Labour Market Studies^10^ held by Statistics Sweden. Individual level data from all registers were linked using the unique personal identity number assigned to all Swedish residents.^11^

Diagnoses in the NPR were classified according to the Swedish version of the 10th revision of the International Statistical Classification of Diseases and Related Health Problems (ICD-10) and date of death from any cause in the CDR were classified according to the 10th revision of the International Classification of Diseases (ICD-10), both since 1997.

The PDR contains data on prescription drugs dispensed at pharmacies since July 2005. Medicinal products are classified using the Anatomical Therapeutic Chemical (ATC) classification system in groups at five distinct levels.^12 13^

All the registers have high completeness and coverage.^5^,^10^ For example, the CDR was initiated in 1952 and has almost 100% completeness for Swedes who die in Sweden or abroad. The PDR is the youngest of the registers, initiated in July 2005 and has 100% coverage of filled prescriptions. Complete register data for the actual period under study, from each one of the registers included was used.

### Derivation of comorbidity indices

The NMI^3^ and the CCI^1^ adapted for register-based research in Sweden^14^ and the ECI^2^ were calculated^15 16^ using in- and out-patient diagnoses identified during look-back periods of one to 5 years before 1 January 2015. For the NMI, dispensed drugs from the 6-month period 1 July to 31 December 2014 were used.^3^

### Derivation of explorative measures

The NPR was used to derive three explorative measures of comorbidity: A, the number of distinct 3-character ICD-10 codes registered as in-patient main diagnoses; B, the number of hospitalisations, that is, in-hospital stays and C, the total length of in-hospital stays. These measures were also calculated using one to 5 years look-back before 1 January 2015.

The PDR was used to derive five explorative measures based on the number of distinct 1-, 3-, 4-, 5- or 7-character ATC codes, respectively, of a person’s all filled prescriptions during the period 1 January to 31 December 2014.

### Outcome

Mortality, that is, date of death from any cause, was retrieved from the CDR.

### Statistical analyses

The discriminatory performance of the comorbidity indices was assessed using the Area Under the Receiver Operating Characteristic curve (AUROC). Models of age-and-sex alone were analysed in all follow-up settings described below and accounted for in all full model analyses, respectively.

We computed the AUROC for mortality at 1, 2, 3, 4 and 5 years of follow-up along with 95% CIs. As the indices and the three simple measures from the in-patient register were measured using five look-back periods of 1, 2, 3, 4 and 5 years of historic data and using the five mortality outcome periods, it was possible to calculate five by five, that is, 25 AUROC for each index and measure explored, respectively. For the five measures explored based on filled prescriptions during the year before 1 January 2015, one AUROC could be calculated for each of the five mortality outcome periods.

Calibration of the complex indices NMI, CCI and ECI, respectively, was assessed over the whole range of values by plotting the proportions of index values within the categorised index groups based on the 5-year look-back period of historic data for prediction of mortality during 5 years of follow-up. Observed 5-year mortality and predicted 5-year mortality with 95% CIs were then compared.

All analyses were performed using SAS V.9.4 (SAS Institute Inc., Cry, NC, USA).

### Ethics

The register data pooled for the CoVacSafe-SE platform is approved by the Swedish Ethical Review Authority (2020-06859, 2021-02186). It has conformed to the principles embodied in the Declaration of Helsinki.

## Results

General characteristics of the 5 010 261 individuals in the study population at baseline are presented in table 1. The mean age was 60 years and 51.4% were women. Malignancies, heart failure or heart flutter and diabetes were the most common comorbid conditions. Cardiovascular preventive drugs, hypnotics or anxiolytics and analgesics were the most common filled prescriptions. The 30 most common causes of mortality during follow-up 2015–2019 and the 30 most common filled prescriptions during 2014, before follow-up are presented in online supplemental appendix tables A1 and A2. There were 441 254 deaths (8.9%) over the 5 years, 2015–2019 (online supplemental appendix table A3).

**Table 1:** Population characteristics for the population at risk on 1 January 2015

Age	Men	Women	Total
Population, n (%)	2 437 016 (48.6)	2 573 245 (51.4)	5 010 261 (100)
Age, median (q1– q3)	59 (49–70)	61 (50–72)	60 (50–71)
Age 40–54, n (%)	946 993 (38.9)	924 066 (35.9)	1 871 059 (37.3)
Age 55–64, n (%)	563 322 (23.1)	561 435 (21.8)	1 124 757 (22.4)
Age 65–79, n (%)	710 917 (29.2)	748 162 (29.1)	1 459 079 (29.1)
Age 80+, n (%)	215 784 (8.9)	339 582 (13.2)	555 366 (11.1)
Medical history, ICD-10 codes, n (%)			
Cerebrovascular disease, I60–I69	76 409 (3.1)	64 886 (2.5)	141 295 (2.8)
Chronic kidney disease, N18, N19	32 453 (1.3)	19 717 (0.8)	52 170 (1.0)
COPD, J44	38 894 (1.6)	46 559 (1.8)	85 453 (1.7)
Dementia, F00–F03, G30	21 960 (0.9)	35 482 (1.4)	57 442 (1.1)
Diabetes, E10–E14	159 849 (6.6)	121 574 (4.7)	281 423 (5.6)
Heart failure, atrial flutter, I50, I48	165 200 (6.8)	125 691 (4.9)	290 891 (5.8)
Ischaemic heart diseases, I20–I25	163 074 (6.7)	98 698 (3.8)	261 772 (5.2)
Malignancies (all types), C00–C97	199 614 (8.2)	192 431 (7.5)	392 045 (7.8)
Thromboembolic disease, I26, I80, I81, I82	39 990 (1.6)	39 976 (1.6)	79 966 (1.6)
Medicines, ATC codes, n (%)			
CV preventive medicines*	908 708 (37.3)	932 838 (36.3)	1 841 546 (36.8)
Proton pump inhibitors, A02B	240 339 (9.9)	349 389 (13.6)	589 728 (11.8)
Drugs for constipation, A06A	116 289 (4.8)	189 293 (7.4)	305 582 (6.1)
Antidiabetic medicines, A10A, A10B	205 249 (8.4)	147 913 (5.7)	353 162 (7.0)
Calcium and vitamin D, A12A	45 356 (1.9)	195 654 (7.6)	241 010 (4.8)
Vitamin B-12, B03B	146 285 (6.0)	232 009 (9.0)	378 294 (7.6)
Thyroid hormones, H03A	57 202 (2.3)	277 431 (10.8)	334 633 (6.7)
Antibiotics, J01	243 717 (10.0)	381 204 (14.8)	624 921 (12.5)
Antirheumatics, M01A	207 303 (8.5)	280 554 (10.9)	487 857 (9.7)
Opioids, N02A	164 228 (6.7)	238 462 (9.3)	402 690 (8.0)
Analgesics, N02B	244 096 (10.0)	452 849 (17.6)	696 945 (13.9)
Antiepileptics, N03A	59 173 (2.4)	75 022 (2.9)	134 195 (2.7)
Antipsychotics, N05A	40 809 (1.7)	52 552 (2.0)	93 361 (1.9)
Hypnotics, anxiolytics, N05C, N05B	241 203 (9.9)	457 873 (17.8)	699 076 (14.0)
Antidepressants, N06A	182 964 (7.5)	374 465 (14.6)	557 429 (11.1)
Adrenergic for asthma, R03A	115 607 (4.7)	178 392 (6.9)	293 999 (5.9)
Country of birth, n (%)			
Swedish-born with two Swedish-born parents	1 655 302 (67.9)	1 645 560 (63.9)	3 300 862 (65.9)
Swedish-born with at least one Swedish-born parent	232 935 (9.6)	240 777 (9.4)	473 712 (9.5)
Swedish-born with foreign-born parents	189 917 (7.8)	277 066 (10.8)	466 983 (9.3)
1st generation immigrants born in Europe	220 633 (9.1)	264 740 (10.3)	485 373 (9.7)
1st generation immigrants born outside Europe	138 229 (5.7)	145 102 (5.6)	283 331 (5.7)

CV preventive medicines*: platelet aggregation inhibitors, B01AC; beta-blockers, C07A; HMG CoA (3-hydroxy-3-methylglutaryl-coenzyme A) reductase inhibitors (statins), C10A; loop-diuretics, C03C; ARBs (angiotensin receptor blockers, including combinations), C09C, C09D; ACE-inhibitors (angiotensin converting enzyme inhibitors), C09A; calcium channel inhibitors, C08C.

A selection of underlying morbidity, that is, 5 years history before 2015 and numbers of prescribed drugs dispensed at pharmacies during 1 July 2014 to 31 December 2014.

ATC, Anatomical Therapeutic Chemical; COPD, chronic obstructive pulmonary disease; ICD-10, 10th revision of the International Classification of Diseases.

The 30 most common filled prescriptions during 2014, before follow-up are presented in online supplemental appendix table 1. There were 441 254 deaths (8.9%) over the 5 years, 2015–2019 as shown in online supplemental appendix table A2. The 30 most common causes of mortality during follow-up are presented in online supplemental appendix table A3.

**Figure 1:**
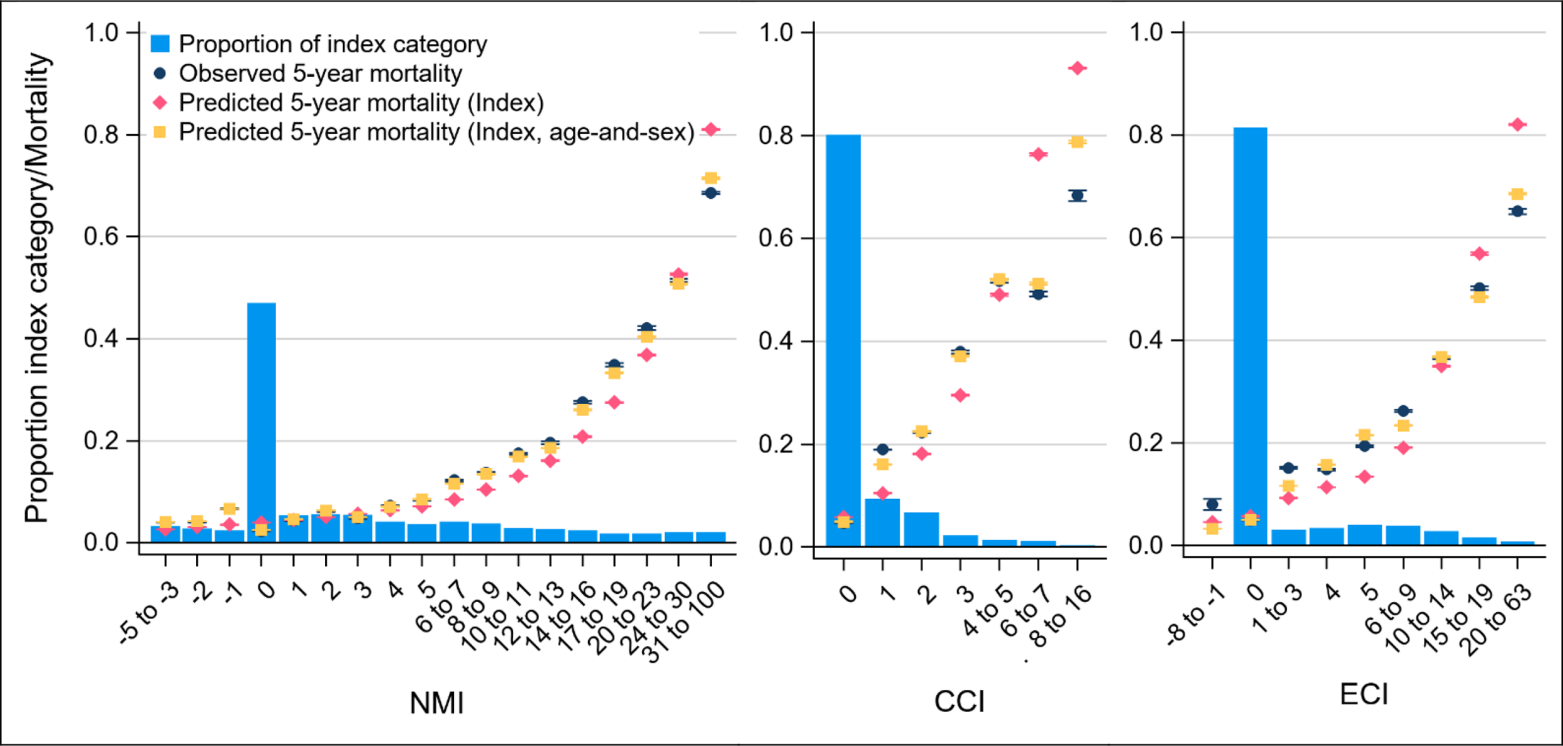
The distribution of the Nordic Multimorbidity Index to the left, the Charlson Comorbidity Index in the middle and the Elixhauser comorbidity index to the right is presented along with observed and predicted 5-year mortality (95% CI) by the level of respective index alone and when added to age-and-sex. CCI, Charlson Comorbidity Index; ECI, Elixhauser Comorbidity Index; NMI, Nordic Multimorbidity Index.

Nearly 50% of the population had a zero-value for the NMI, and 80% had a zero-value for the CCI and the ECI, respectively (figure 1). The NMI and ECI take on a much broader range of values than the CCI and the proportion of non-zero values for the NMI and the ECI was more evenly distributed than that of CCI, where a majority had a non-zero value of either ‘1’ or ‘2’. For the NMI and the ECI, respectively, when added to age-and-sex, predictions were very close to observed mortality, that is, a good calibration, over the whole range of index values (figure 1) whereas the CCI when added to age-and-sex, overestimated mortality risk at values around six and above. In online supplemental appendix table A4 the distribution of subcomponents of the NMI and their weights are presented.

**Figure 2:**
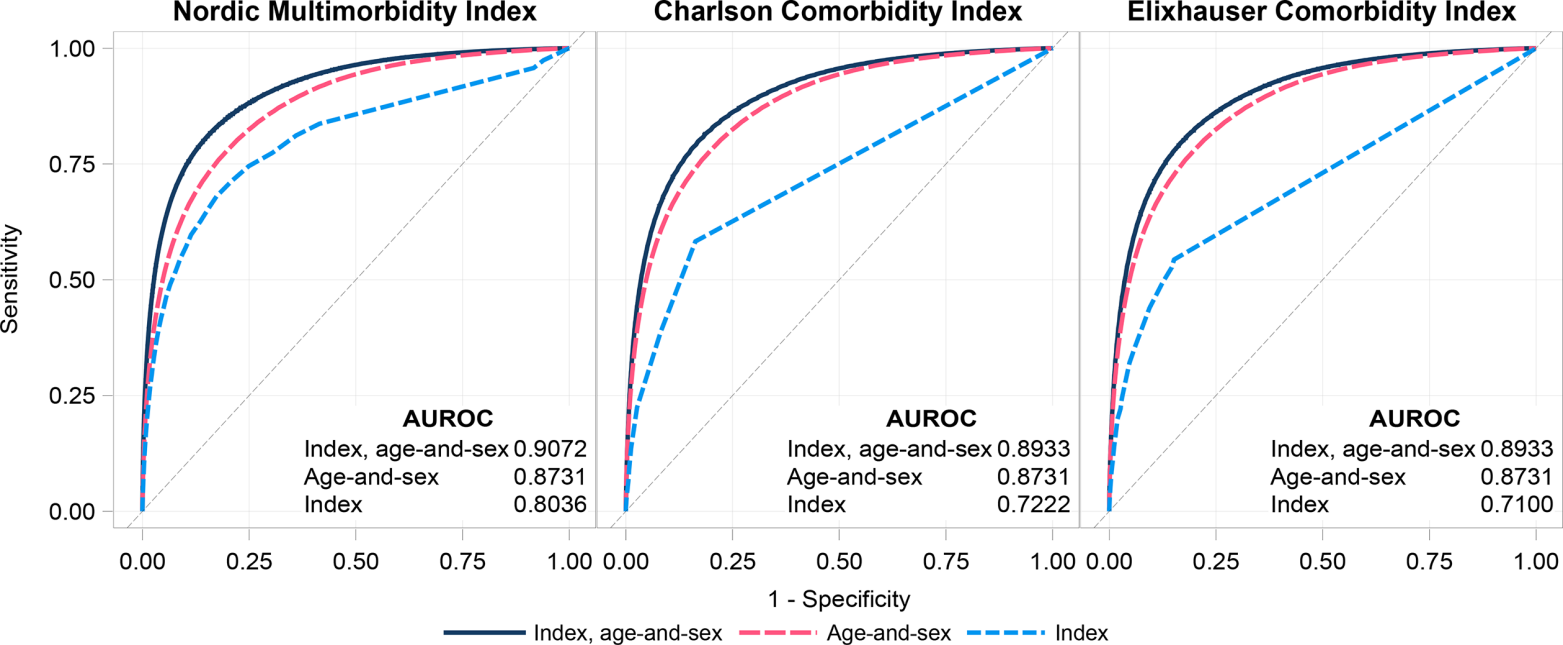
Area under the Receiver Operating Curve characteristics for 5-year mortality with a 5-year look-back period for diagnoses, the Nordic Multimorbidity Index to the left and the Charlson Comorbidity Index to the right. Models based on (a) age-and-sex, (b) NMI and CCI alone and (c) age-and-sex *and* NMI and CCI, respectively. AUROC, Area Under the Receiver Operating Characteristic curve; CCI, Charlson Comorbidity Index; NMI, Nordic Multimorbidity Index.

The AUROC for age-and-sex alone was 0.8731 for 5-year mortality, with a 5-year look-back period. For the NMI alone the AUROC was 0.8036 and for the CCI and the ECI 0.7222 and 0.7100, respectively (figure 2). When age-and-sex was added to the respective indices, the AUROC for the NMI increased to 0.9072 and for both the CCI and the ECI to 0.8933, that is, no difference up to the fourth decimal place between CCI and ECI. With age-and-sex added the absolute difference between the NMI and the CCI and ECI, respectively, was 0.0139 (95% CI 0.0137 to 0.0141).

**Figure 3:**
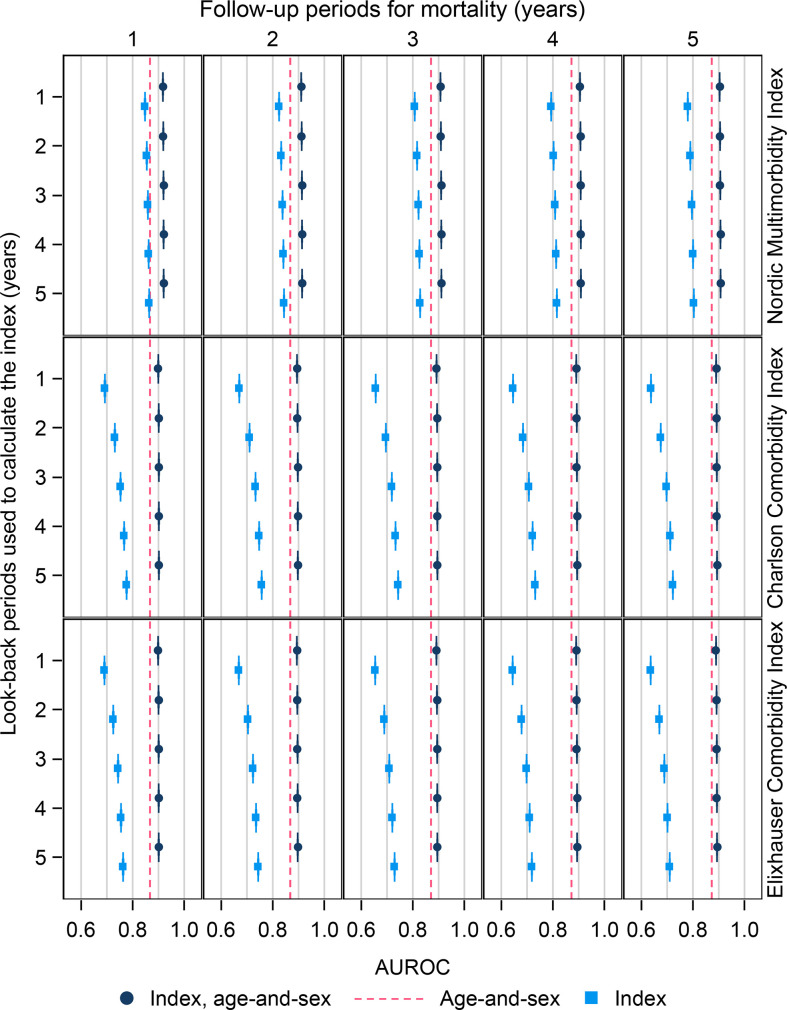
Area under the Receiver Operating Curve characteristics for 1- to 5-year mortality with a 1- to 5-year look-back period for diagnoses for the Nordic Multimorbidity Index (top), the Charlson Comorbidity Index (middle) and Elixhauser Comorbidity Index (bottom). Models based on (a) age-and-sex, (b) index alone and (c) age-and-sex *and* index. AUROC, Area Under the Receiver Operating Characteristic curve,

For models where NMI was added to age-and-sex, a consistent pattern was observed with an AUROC marginally separated from 0.91 irrespective of length of follow-up or look-back period, 1–5 years respectively, however age-and-sex alone were very close to the full models (figure 3). For both the CCI alone and the ECI alone, the shorter the look-back period, the lower the AUROC was observed, irrespective of follow-up period. In general, full models of CCI and ECI, respectively, when added to age-and-sex were only marginally better than age-and-sex alone.

**Figure 4:**
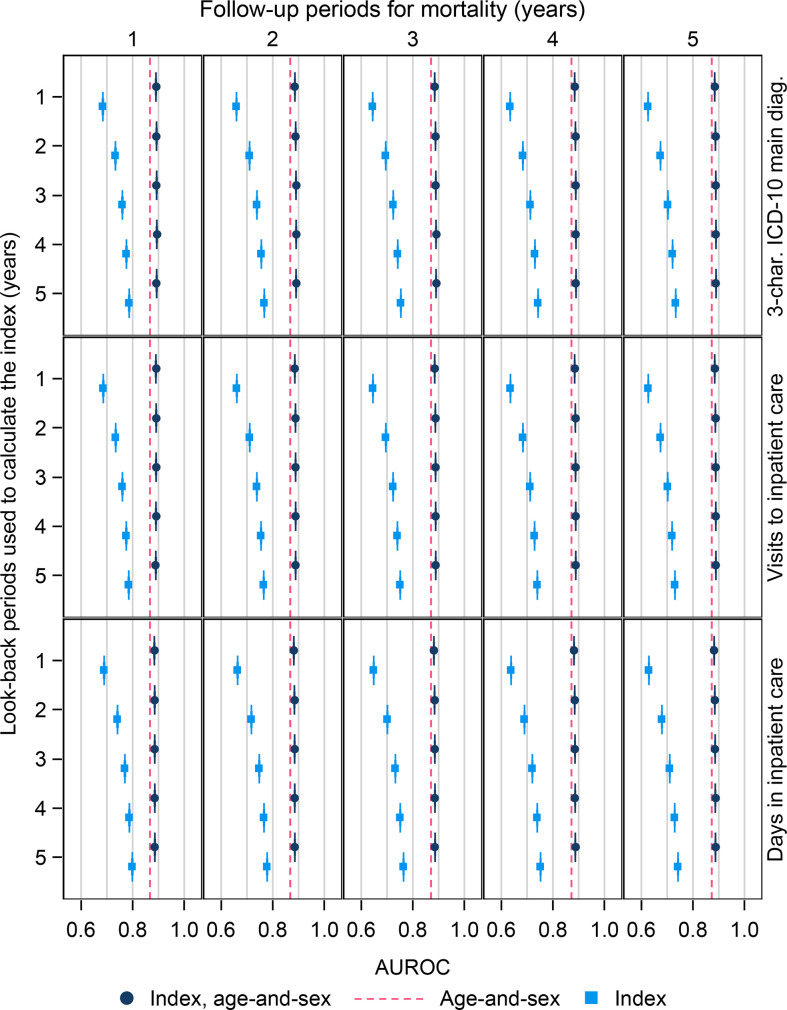
Area under the Receiver Operating Curve characteristics for 1- to 5-year mortality with a 1- to 5-year look-back period for the following measures: numbers of distinct 3-character ICD-10 codes of main diagnoses (top), numbers of visits (middle) and numbers of days hospitalised for in-patient care (bottom). Models based on (a) age-and-sex, (b) measure alone and (c) age-and-sex *and* measure. AUROC, Area Under the Receiver Operating Characteristic curve; ICD-10, 10th revision of the International Classification of Diseases.

Models of measures of in-patient care utilisation, when added to age-and-sex, yielded AUROC marginally separated from 0.90 irrespective of length of follow-up or look-back period and were marginally better than age-and-sex alone (0.87) (figure 4). For measures of in-patient care utilisation alone, the AUROC increased from around 0.60 to 0.75 when look-back periods were increased from 1- to 5 years, and AUROC decreased slightly with increasing follow-up time, but were still much lower than AUROC for age-and-sex alone.

For models of measures of filled prescriptions, when added to age-and-sex, with a 1-year look-back period yielded an AUROC scanty below 0.89 irrespective of length of follow-up and were marginally better than age-and-sex alone (0.87) (online supplemental appendix figure A1). For the measures of filled prescriptions alone, the AUROC decreased from slightly above to slightly below 0.80 when follow-up periods increased from 1 to 5 years, that is, much lower than age-and-sex alone.

In the youngest age group of 40-plus, the explorative measures alone, of distinct 1-, 3-, 4-, 5- and 7-character ATC codes showed larger AUROC than CCI, ECI and the numbers of ICD-10 (distinct 3-character) diagnoses, numbers of hospitalisations or days hospitalised. However, the opposite pattern was found in the oldest age group of 80-plus where the distinct ATC codes showed lower AUROC than CCI, ECI and the numbers of ICD-10 (distinct 3-character) diagnoses, numbers of hospitalisations or days hospitalised. However, the NMI alone showed largest AUROC in all three age groups.

Online supplemental appendix table A4 includes proportions of respective subcomponent of the NMI in the study populations and weights applied from the original publication.^3^ Further, the appendix includes online supplemental tables A5–A8 on numerical results on analyses presented in figures 3 and 4 and online supplemental appendix figures A1 and A2 for full transparency as the very narrow 95% CIs in the figures might be difficult to assess without greatly enlarging the figures.

## Discussion

In this study, we observed that adding NMI to age-and-sex marginally improved the ability to discriminate for mortality as compared with age-and-sex alone. When adding CCI or ECI to age-and-sex less improvement was observed. Similarly, for the explorative measures, that is, numbers of diagnoses, numbers of hospitalisations, numbers of days in in-patient care or filled prescriptions, when added to age-and-sex, the AUROCs were marginally increased as compared to age-and-sex alone. This is not that surprising given that age and sex are powerful predictors of mortality. Thus, an AUROC near 0.9 was observed in all models of the NMI, CCI or ECI indices or any of the explorative measures when added to age-and-sex implicating the large impact of age on the models. Further, we observed similar findings in all settings with one to 5 years of historic data used and for all follow-up periods of 1–5 years for mortality. However, all indices and explorative measures, as well as age-and-sex alone, performed relatively worse the older the age group. This phenomenon is due to a lower incremental value added of an increase to a score in an older age group where co-morbid conditions are more prevalent. Fewer old individuals are without comorbidities, that is, zero-level scores are less prevalent and scores in older age groups are on the average higher. In contrast, in younger individuals’ comorbid conditions are fewer, zero-level scores are frequent, and scores are on the average lower. Comorbidity is thus a stronger marker for mortality in a younger age group. Anyway, age-and-sex should always be used routinely as covariates in regression models when using registry data. Furthermore, the explorative measures reflecting health care consumption as numbers of diagnoses, hospitalisations, days hospitalised or filled prescriptions could be considered valid alternatives, especially in situations where full data are lacking, see further below.

The NMI was developed to be specifically suited to the comprehensive and population-wide health- and administrative registries available in the Nordic countries^3^ with individual level data on, among others, hospital diagnoses and use of filled prescriptions. Since the CCI and ECI were developed on hospitalised patients in the USA in 1987 and 1998, respectively, diagnostic practices and prognosis of many diseases have changed substantially limiting the generalisability of these older indices in today’s research. As examples, treatment options available have improved cancer survival and HIV prognosis. Further, mortality rates in CVDs have decreased.^17^ It is possible that changes with time in prognosis and improved treatments of diseases have contributed to the observed differences between the NMI and the CCI or ECI in performance as the respective weights of the CCI and ECI were derived some 30 years before our baseline. Further, the populations used for deriving the NMI, the CCI and the ECI, respectively, differ regarding age, ethnicity, prevalence of chronic diseases and most importantly in mortality rates. The NMI is intended to predict mortality for the general population 40 years and older, whereas the CCI and ECI were developed to predict mortality for hospitalised patients. The performance of the NMI in the present study of a general population was better as compared with the CCI and ECI regarding discriminative ability even though observed differences in AUROC appear to be small.

The NMI includes information on filled prescriptions of drugs in addition to diagnosis codes from hospitalisations and out-patient visits in specialised care, while the CCI and ECI are only based on diagnosis codes from hospitalisations. Filled prescriptions data are readily available in health registries in all Nordic countries. By including filled prescriptions of drugs, the NMI also captures conditions treated in the primary care setting outside hospitals. This may contribute to the small increase observed in predictive performance of the NMI as compared with CCI and ECI. Further, high proportions of individuals in our general population had CCI=0 and/or ECI=0, indicating no comorbidity which possibly may contribute to poorer performance of these two indices. Furthermore, when restricting the population to older age groups, the AUROC for age-and-sex alone was diminishing, which is to be expected as the distribution of age is narrowed. Despite higher mortality among the oldest, their more frequent co-morbidity reflects a greater variety and severity of illness, thus representing a ‘more diffuse nature’ in the register data and other aspects of health affecting the outcome is lacking. Thus, these aspects contribute to the poorer performance in this group as compared with the youngest age group, where previous disease at baseline more distinctly, that is, with higher precision discriminates between a diseased and a healthy person. The NMI when added to age-and-sex showed the best performance as compared with CCI or ECI or any of the explorative measures in the oldest group, 80-plus years with an AUROC of 0.80 as seen in figure 4 implying that it is the most useful measure of comorbidity for use in registry studies in older age groups.

The explorative measures based on quantifying in-patient care showed, respectively, better discriminative performance the longer look-back used when not added to age-and-sex, that is, higher levels of historic data and/or granularity improve discrimination. However, for models of these measures, respectively, when added to age-and-sex, AUROC for 1- to 5-year mortality, was around 0.9 irrespectively of length of the look-back period indicating the large impact on AUROC of information on age-and-sex. However, numbers of days hospitalised, when added to age-and-sex performed as well as the NMI, CCI or ECI in our general population above 40 years of age. Interestingly, when analysing large datasets, using variables agnostic to their meaning can improve predictive ability as shown in a recent study of 10-year mortality where men selected as controls to prostate cancer patients showed that comorbidity assessment can be improved by use of all ICD-10 codes and taking related frequency, recency and duration of hospital admissions into account.^18^

For the explorative measures based on filled prescriptions, only minimal differences were observed for the number of distinct characters of ATC codes used in all models of these measures for 1- to 5-year mortality, respectively, when added to age-and-sex. Thus, the discrimination for mortality increased only marginally as compared with age-and-sex alone indicating the large impact of age-and-sex on AUROC in these models. Further, numbers of distinct 4-, 5- or 7-character ATC codes alone performed similarly to each other but better than 1- or 3-character ATC codes or the CCI or ECI alone in our general population above 40 years as seen in figure 6. This indicates that a lower than full level of ATC-code granularity, that is, the 4-character ATC-code level covers the essential data needed. However, in general, the ATC codes alone performed less effective in the age group 65 plus and less so in the highest age group of 80 plus. All in all, the NMI alone or when added to age-and-sex showed the highest AUROC of all indices or measures analysed in all three age groups.

For the NMI a look-back period of 6 months for filled prescriptions was used in the original study on NMI.^3^ The 1-year look-back period for extracting diagnoses from the national patient register rendered similar AUROC as a 5-year look-back. Thus, the NMI may perform well when only a short look-back period is available. However, as commonly practised in Sweden, some chronic diseases are predominantly treated in primary care after a hospitalisation some years back. Thus, short look-back data might miss some chronic diseases that longer look-back period may better capture the impact of. As observed, the NMI can take on negative values (see figure 1) as the weights for some ATC codes are negative. This may not necessarily be interpreted as a causal protective effect of drugs used for primary prevention purposes. It may as well reflect prescription practices in patients with perceived expectancy of longer life and thus indirectly represent aspects of better health.

The NMI was developed using Danish registry data with the purpose of providing a summary score to adjust for confounding from comorbidity in register-based research. Even if the use of known confounders should be preferred, a comorbidity score may be useful when high-dimensional approaches in analyses are unfeasible. It can act as a substitute for the individual comorbidity variables for confounding control in epidemiological research and reduce the number of variables that are included in a model allowing for adjustments of more confounding variables when analysing smaller datasets and thus handling problems with possible risk of overfitting.^19^

### Strengths and limitations

Major strengths of the present study were the use of nationwide data on hospital diagnoses and filled prescriptions, from national health data registers with high completeness, coverage and validity^7^ including the whole Swedish population aged 40 years and older with complete information on mortality.

Meaningful quantification of comorbidity is complex. The advantage of the NMI is that it is derived using in- and out-patient diagnosis data from specialised care and filled prescriptions from both specialised and primary care and thus has a lower threshold for defining comorbidity compared to using smaller sets derived from hospitalisation data alone. However, adding NMI to the powerful predictors of age-and-sex for death only marginally improved the ability to discriminate mortality, compared to age-and-sex alone. This was also observed for exploratory measures. The large population sample gave us the opportunity to perform analyses in subgroups of older age. The higher the age group, the lower the AUROC, implying reduced usability in studies of older patients, where comorbidity is more common and thus less discriminatory. Generalisability of the results depends on the setting. We assume that results are representative to countries with similar systems for health care and health registries. Extrapolation of results to later dates than end of follow-up by the end of 2019 is reasonable. The time frame was chosen to not be affected by the COVID-19 pandemic, which started in 2020 as we cannot exclude that analyses of relations between indices and mortality may be affected.

### Future perspectives

Future research should also look at NMI and explorative measures performance when using disease diagnoses and conditions identified from primary care data. Such register data will most probably be available in the future in Sweden as well as a register on drugs administered during hospitalisation as new legislation is under development. Thus, there will be increasing information on use of pharmaceutical products where the performance of explorative measures using ATC-code data should be further explored in a widened register landscape. The inclusion of data on laboratory values is not possible as such data at present are lacking in the national health registers, However, future analyses of performance of a wider array of explorative measures could include using large-scale European data sources.^20^ Given that some chronic conditions would be expected to have strongly differential impact on other outcomes than mortality it is likely that separately developed targeted indices may still perform better than the NMI, CCI or ECI for considering the impact of comorbidity on outcomes such as different cancers or cardiovascular diseases. Thus, NMI and measures as the numbers of days hospitalised or distinct 4-character ATC-codes in combination with age-and-sex and key ICD-10 and or ATC code information should be further explored in their abilities to control for confounding in observational research with other outcomes than mortality. Furthermore, ATC-codes should also be further explored using cluster-based score analyses, helping in identifying meaningful, reproducible markers for multiple disease groupings.

## Conclusion

In all models analysed using population based Swedish registry data, without including age-and-sex in the model, the NMI showed improved discrimination compared to the CCI and ECI, respectively, in all settings when using shorter and longer look-back period of historical data on both 1- and 5-year mortality as the outcome. However, age-and-sex alone performed better than NMI, CCI or ECI alone. Thus, the NMI provides modest additional discrimination, with CCI and ECI both even less helpful, that is, informative.

However, while the NMI combined with age-and-sex summarises comorbidity well the surrogate measures of days hospitalised or numbers of distinct 4-character ATC-codes, respectively, when added to age-and-sex performed nearly as well as NMI in terms of AUROC.

## Supplementary material

10.1136/bmjopen-2025-114635 online supplemental file 1

## Data Availability

Data are available upon reasonable request. Data may be obtained from a third party and are not publicly available.
