# Supplementary material for: Nordic Multimorbidity Index, Charlson, Elixhauser and count-based comorbidity indices for mortality risk: a comparative nationwide cohort study using national health registers in Sweden
Source: BMJ Open. 2026 Jun 22;16(6):e114635. doi: 10.1136/bmjopen-2025-114635 (PMC13288703; doi:10.1136/bmjopen-2025-114635)

**APPENDIX**

The Nordic Multimorbidity Index, Charlson, Elixhauser and other indices of comorbidity for mortality risk. Comparative nationwide cohort study using national health register data in Sweden

Report from the Swedish Medical Products Agency

By Björn Zethelius et al.

**Table of contents**

Table and figure legends below are truncated

**Table A1** Listing of the 30 most frequent causes of death 2010-2014

**Table A2** Listing of the 30 most frequent prescribed drugs dispensed, July to December 2014

**Table A3** Mortality in the population at risk 2010-2014, cumulative up to five years

**Table A4** Proportions of the respective subcomponents of the Nordic Multimorbidity Index

**Figure A1** AUROC for mortality for numbers of distinct 1-, 3-, 4-, 5- and 7-character ATC-codes

**Figure A2** AUROC for mortality in three age groups (years) 40-plus; 65-plus and 80-plus

**Table A5** AUROC for mortality Numerical results corresponding to Figure 3

**Table A6** AUROC for mortality Numerical results corresponding to Figure 4

**Table A7** AUROC for mortality Numerical results corresponding to Appendix Figure A1

**Table A8** AUROC for mortality Numerical results corresponding to Appendix Figure A2

**Table A1.** Listing of the 30 most frequent (presence 6.7 – 0.8%) underlying causes of death during the follow-up period 2015- 2019. ICD-10-codes (3-positions).

| **ICD-10** | **Frequency** | **%** | **Diagnosis** |
| --- | --- | --- | --- |
| I25 | 29 440 | 6,7 | Chronic Ischemic Heart disease |
| I21 | 23 930 | 5,4 | Acute Myocardial Infarction |
| F03 | 23 033 | 5,2 | Unspecified Dementia |
| C34 | 17 992 | 4,1 | Malignant Neoplasm of bronchus and Lung |
| I50 | 16 395 | 3,7 | Heart Failure |
| G30 | 15 205 | 3,4 | Alzheimer's Disease |
| J44 | 14 079 | 3,2 | Chronic obstructive pulmonary disease |
| I48 | 13 930 | 3,2 | Atrial Fibrillation and Flutter |
| C61 | 11 528 | 2,6 | Malignant neoplasm of prostate |
| C18 | 9 121 | 2,1 | Malignant neoplasm of colon |
| C25 | 9 085 | 2,1 | Malignant neoplasm of pancreas |
| J18 | 8 870 | 2,0 | Pneumonia, unspecified organism |
| I64 | 7 672 | 1,7 | Acute cerebrovascular disease, unspecified bleeding or infarction |
| F01 | 7 152 | 1,6 | Pneumonia, unspecified organism |
| C50 | 6 793 | 1,5 | Malignant neoplasms of breast |
| R99 | 6 754 | 1,5 | Ill-defined and unknown cause of Mortality |
| I11 | 6 307 | 1,4 | Hypertensive heart disease |
| E14 | 6 090 | 1,4 | Diabetes Mellitus, unspecified |
| I69 | 6 061 | 1,4 | Sequelae of cerebrovascular disease |
| I63 | 5 748 | 1,3 | Cerebral infarction |
| C80 | 5 376 | 1,2 | Lymphoma, unspecified |
| I61 | 4 978 | 1,1 | Nontraumatic intracerebral haemorrhage |
| X59 | 4 756 | 1,1 | Injury, unspecified |
| R54 | 4 541 | 1,0 | Age-related physical debility |
| I71 | 4 159 | 0,9 | Aortic aneurysm and dissection |
| A41 | 4 074 | 0,9 | Sepsis, unspecified organism |
| I35 | 3 981 | 0,9 | Nonrheumatic aortic valve disorders |
| G20 | 3 915 | 0,9 | Parkinson's disease |
| C20 | 3 793 | 0,9 | Malignant neoplasm of rectum |
| E11 | 3 716 | 0,8 | Type 2 diabetes mellitus |
| Other | 152 780 | 34,6 | Other underlying causes of death |
| All | 441 254 | 100,0 | All underlying causes of death |

Abbreviations: ICD-10-codes denotes International Classifications of Disease, 10^th^ revision

**Table A2.** Listing of the 30 most frequent (presence 4.3 – 1.0%) distinct 4-character ATC codes of prescribed drugs dispensed at pharmacies during January 1^st^, 2014, to December 31^st^, 2014, from which a selection was presented in table 1.

| **ATC-4** | **Frequency** | **%** | **Type of medicine** |
| --- | --- | --- | --- |
| N02B | 919 012 | 4,3 | Analgesics paracetamol |
| B01A | 916 250 | 4,3 | Platelet aggregantia inhibitors |
| C07A | 905 810 | 4,2 | Betablockers |
| C10A | 844 973 | 4,0 | HMG CoA reductase inhibitors (statins) |
| M01A | 765 125 | 3,6 | Antirheumatic drugs |
| A02B | 729 850 | 3,4 | Proton pump inhibitors |
| J01C | 687 751 | 3,2 | Betalactam antibiotics, Penicillin |
| N05C | 650 299 | 3,0 | Hypnotics and sedatives |
| N06A | 633 828 | 3,0 | Antidepressants |
| C08C | 607 476 | 2,8 | Calcium channel blockers |
| N02A | 581 444 | 2,7 | Opioids |
| C09A | 557 211 | 2,6 | ACE inhibitors |
| A06A | 444 181 | 2,1 | Drugs for constipation |
| B03B | 430 166 | 2,0 | Vitamin B12 and Folate |
| C09C | 429 364 | 2,0 | ARB and A2 blockers |
| N05B | 402 602 | 1,9 | Anxiolytics |
| R03A | 401 588 | 1,9 | Adrenergics for Bronchial asthma |
| D07A | 375 246 | 1,8 | Corticosteroids |
| R06A | 353 355 | 1,7 | Antihistamines |
| H03A | 347 778 | 1,6 | Thyroid hormones |
| H02A | 328 844 | 1,5 | Corticosteroids for systemic use |
| G03C | 324 669 | 1,5 | Sex hormones |
| R01A | 322 354 | 1,5 | Nasal preparations, anti congestants |
| R05C | 304 551 | 1,4 | Expectorants and mucolytics |
| C03C | 288 871 | 1,4 | Loop diuretics |
| A10B | 288 393 | 1,4 | Antidiabetic medicines |
| A12A | 283 867 | 1,3 | Calcium channel blockers |
| D02A | 275 178 | 1,3 | Emollients Moisturizer |
| R03B | 256 090 | 1,2 | Inhalants excluding beta 2 stimulators |
| R05F | 253 368 | 1,2 | Expectorants and mucolytics |
| Other | 6 414 833 | 30,1 | Other types of medicines |
| All | 21 324 327 | 100,0 | All filled prescriptions |

Abbreviations: HMG CoA denotes 3-hydroxi-3-methylglutaryl-coenzyme A; ACE, Angiotensin Converting Enzyme; ARB, Angiotensin Receptor Blocker; A2, Angiotensin Receptor-2 Blocker.

**Table A3**. Mortality in the population at risk, cumulative up to five years after January 1^st^, 2015.

| **Mortality** n (%) | **Men** | **Women** | **Total** |
| --- | --- | --- | --- |
| Population, | 2,437,016 (48.6) | 2,573,245 (51.4) | 5,010,261 (100) |
| Mortality 1-year | 42,946 (1.8) | 45,634 (1.8) | 88,580 (1.8) |
| Mortality 2-years | 85,764 (3.5) | 91,327 (3.6) | 177,091 (3.5) |
| Mortality 3-years | 128,845 (5.3) | 137,501 (5.4) | 266,346 (5.3) |
| Mortality 4-years | 172,328 (7.1) | 183,233 (7.2) | 355,561 (7.1) |
| Mortality 5-years | 214,365 (8.9) | 226,889 (8.9) | 441,254 (8.9) |
| Abbreviations: n denotes numbers |  |  |  |

**Table A4**. The proportion of the study population with the respective subcomponents of the Nordic Multimorbidity Index, expressed as a percentage with 95% confidence intervals. Data from the in-patient and out-patient registers from 2010 to 2014 and the prescribed drug register from 1 July to 31 December 2014.

| **Weight, subcomponents** | | **Men** | | | **Women** | | |
| --- | --- | --- | --- | --- | --- | --- | --- |
|  |  | **%** | 95% LCL | 95% UCL | **%** | 95% LCL | 95% UCL |
|  | **In- and out-patient registers 2010-2014** |  |  |  |  |  |  |
| 22 | Secondary malignant neoplasms and malignancy of unspecified site | **0.68** | 0.67 | 0.69 | **0.93** | 0.92 | 0.94 |
| 19 | Malignant neoplasm of bronchus and lung | **0.16** | 0.16 | 0.17 | **0.21** | 0.20 | 0.21 |
| 13 | Alcoholic liver disease, liver fibrosis, cirrhosis, and failure | **0.26** | 0.25 | 0.26 | **0.22** | 0.21 | 0.22 |
| 12 | Mental and behavioural disorders due to use of alcohol | **2.15** | 2.13 | 2.16 | **0.91** | 0.90 | 0.92 |
| 11 | Decubitus ulcer and pressure area | **0.16** | 0.15 | 0.16 | **0.16** | 0.16 | 0.17 |
| 10 | Chronic viral hepatitis | **0.61** | 0.60 | 0.62 | **0.34** | 0.33 | 0.34 |
| 9 | Dementia | **0.90** | 0.89 | 0.91 | **1.38** | 1.36 | 1.39 |
| 8 | Leukaemia | **0.24** | 0.23 | 0.24 | **0.17** | 0.16 | 0.17 |
|  | Malignant neoplasm of bladder | **0.62** | 0.61 | 0.63 | **0.20** | 0.19 | 0.20 |
|  | Tumour of brain or meninges | **0.28** | 0.28 | 0.29 | **0.44** | 0.43 | 0.45 |
| 7 | Chronic kidney disease and unspecified kidney failure | **1.33** | 1.32 | 1.35 | **0.77** | 0.76 | 0.78 |
|  | Multiple sclerosis | **0.17** | 0.17 | 0.18 | **0.39** | 0.39 | 0.40 |
|  | Other interstitial pulmonary diseases | **0.16** | 0.15 | 0.16 | **0.13** | 0.12 | 0.13 |
|  | Parkinson’s disease and other parkinsonism | **0.49** | 0.48 | 0.50 | **0.35** | 0.34 | 0.36 |
| 6 | Volume depletion | **0.43** | 0.42 | 0.44 | **0.53** | 0.52 | 0.54 |
| 5 | Anaemia | **2.12** | 2.10 | 2.13 | **3.10** | 3.08 | 3.12 |
|  | Atherosclerosis, thrombosis, embolism, and other peripheral arterial disease | **1.13** | 1.11 | 1.14 | **1.01** | 1.00 | 1.02 |
|  | Diseases of teeth and supporting structures | **0.45** | 0.44 | 0.46 | **0.45** | 0.45 | 0.46 |
|  | Epilepsy | **0.89** | 0.88 | 0.90 | **0.78** | 0.77 | 0.79 |
|  | Malignant neoplasm of prostate | **3.16** | 3.14 | 3.18 | **-** | - | - |
| 4 | Aneurysm and dissection of aorta and other arteries | **0.98** | 0.97 | 1.00 | **0.30** | 0.29 | 0.31 |
|  | Cerebrovascular disease | **3.14** | 3.11 | 3.16 | **2.52** | 2.50 | 2.54 |
|  | Chronic lower respiratory diseases and failure | **1.85** | 1.83 | 1.86 | **2.09** | 2.08 | 2.11 |
|  | Heart failure | **2.95** | 2.92 | 2.97 | **2.27** | 2.25 | 2.29 |
|  | Malignant neoplasm of breast | **0.03** | 0.02 | 0.03 | **1.99** | 1.97 | 2.01 |
|  | Mental and behavioural disorders due to use of tobacco | **0.68** | 0.67 | 0.69 | **0.54** | 0.53 | 0.55 |
|  | Pneumonia | **2.53** | 2.51 | 2.55 | **2.42** | 2.40 | 2.44 |
| 2 | Aortic and mitral valve disease | **1.51** | 1.49 | 1.52 | **1.19** | 1.18 | 1.20 |
|  | Type 2 diabetes mellitus | **5.76** | 5.73 | 5.79 | **4.11** | 4.09 | 4.14 |
|  | **Prescribed drug register 1st July to 31st December 2014** |  |  |  |  |  |  |
| 11 | Anti-dementia drugs | **0.65** | 0.64 | 0.66 | **0.98** | 0.97 | 0.99 |
| 8 | Drugs for constipation | **4.77** | 4.75 | 4.80 | **7.36** | 7.32 | 7.39 |
| 7 | Antipsychotics | **1.54** | 1.52 | 1.55 | **1.85** | 1.84 | 1.87 |
|  | Drugs used in opioid dependence | **0.09** | 0.08 | 0.09 | **0.06** | 0.05 | 0.06 |
| 5 | Antipropulsives | **0.66** | 0.65 | 0.67 | **0.88** | 0.87 | 0.90 |
|  | High-ceiling diuretics | **4.28** | 4.25 | 4.30 | **5.60** | 5.58 | 5.63 |
|  | Iron preparations | **0.92** | 0.91 | 0.94 | **1.94** | 1.93 | 1.96 |
|  | Long-acting anti-muscarinic agents | **1.31** | 1.30 | 1.33 | **1.72** | 1.70 | 1.73 |
| 4 | Digitalis glycosides | **0.74** | 0.73 | 0.75 | **0.79** | 0.78 | 0.80 |
|  | Insulins and analogues | **3.64** | 3.62 | 3.67 | **2.50** | 2.48 | 2.52 |
| 3 | Aldosterone antagonists | **1.41** | 1.40 | 1.43 | **1.60** | 1.59 | 1.62 |
|  | Antidepressants | **7.51** | 7.47 | 7.54 | **14.55** | 14.51 | 14.60 |
|  | Short-acting beta agonists | **2.67** | 2.65 | 2.69 | **4.10** | 4.08 | 4.13 |
| 2 | Anilides | **10.00** | 9.97 | 10.04 | **17.58** | 17.53 | 17.63 |
|  | Glucocorticoids for systemic use | **3.54** | 3.52 | 3.56 | **5.10** | 5.08 | 5.13 |
|  | Opioids | **6.74** | 6.71 | 6.77 | **9.27** | 9.23 | 9.30 |
|  | Platelet aggregation inhibitors excluding heparin | **14.04** | 14.00 | 14.09 | **10.87** | 10.83 | 10.90 |
| 1 | Benzodiazepines and related drugs | **8.15** | 8.12 | 8.19 | **15.09** | 15.04 | 15.13 |
|  | Beta-lactam antibacterials, penicillin’s | **6.16** | 6.13 | 6.19 | **9.50** | 9.46 | 9.53 |
| -2 | ARBs including combinations | **11.04** | 11.00 | 11.08 | **10.84** | 10.81 | 10.88 |
| -3 | HMG CoA reductase inhibitors (statins) | **17.66** | 17.61 | 17.70 | **12.90** | 12.86 | 12.94 |

Abbreviations: LCL denotes Lower Confidence Interval Level and UCL denotes Upper Confidence Interval Level; ARB denotes Angiotensin Receptor Blocker; HMG CoA denotes 3-hydroxi-3-methylglutaryl-coenzyme A

**Appendix Figure A1**. Area under the Receiver Operating Curve characteristics for 1- to 5-year mortality with a 1-year look-back period for the following measures: numbers of distinct 1-, 3-, 4-, 5- and 7-character ATC-codes (top to bottom) on filled prescriptions from the prescribed drug register. Models based on (a) age-and-sex, (b) measure alone, and (c) age-and-sex *and* measure.

| 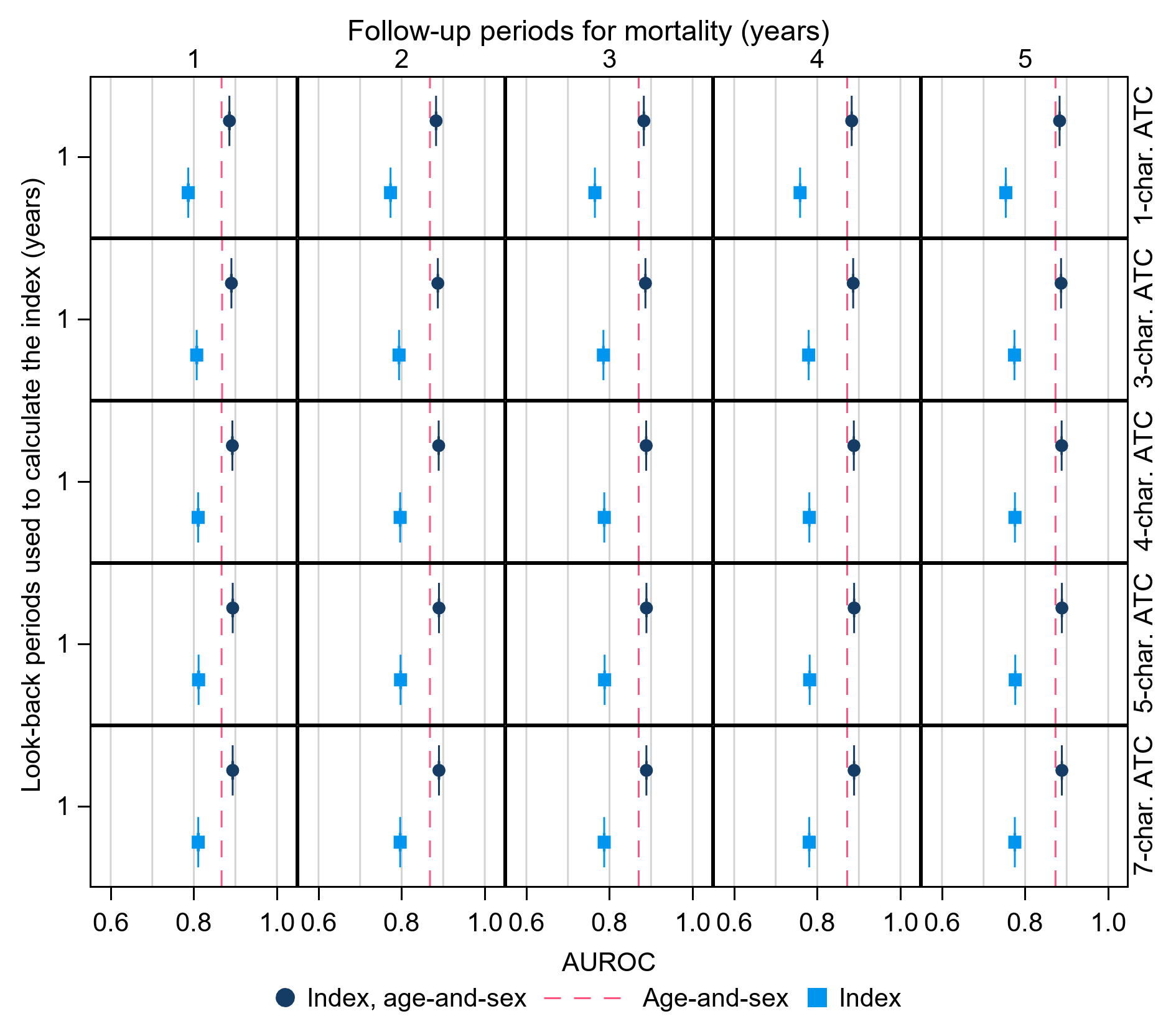 |
| --- |

**Appendix Figure A2**. Area under the Receiver Operating Curve characteristics for 5-year mortality in three specified age groups (years) 40-plus (top); 65-plus (middle); and 80-plus (bottom) with a 5-year look-back period for diagnoses and a 1-year look-back period for filled prescriptions (From top to bottom in each panel) for: The Nordic Multimorbidity Index, the Charlson Comorbidity Index; the Elixhauser Comorbidity Index; numbers of distinct 3-character ICD-10 codes of main diagnoses; numbers of hospitalizations; numbers of days hospitalized for in-patient care; and distinct 1-, 3-, 4-, 5- and 7-character ATC-codes. Models based on (a) age-and-sex (The reference line in respective panel), (b) indices and measures alone to the left, and (c) age-and-sex *and* indices and measure, respectively to the right.

| **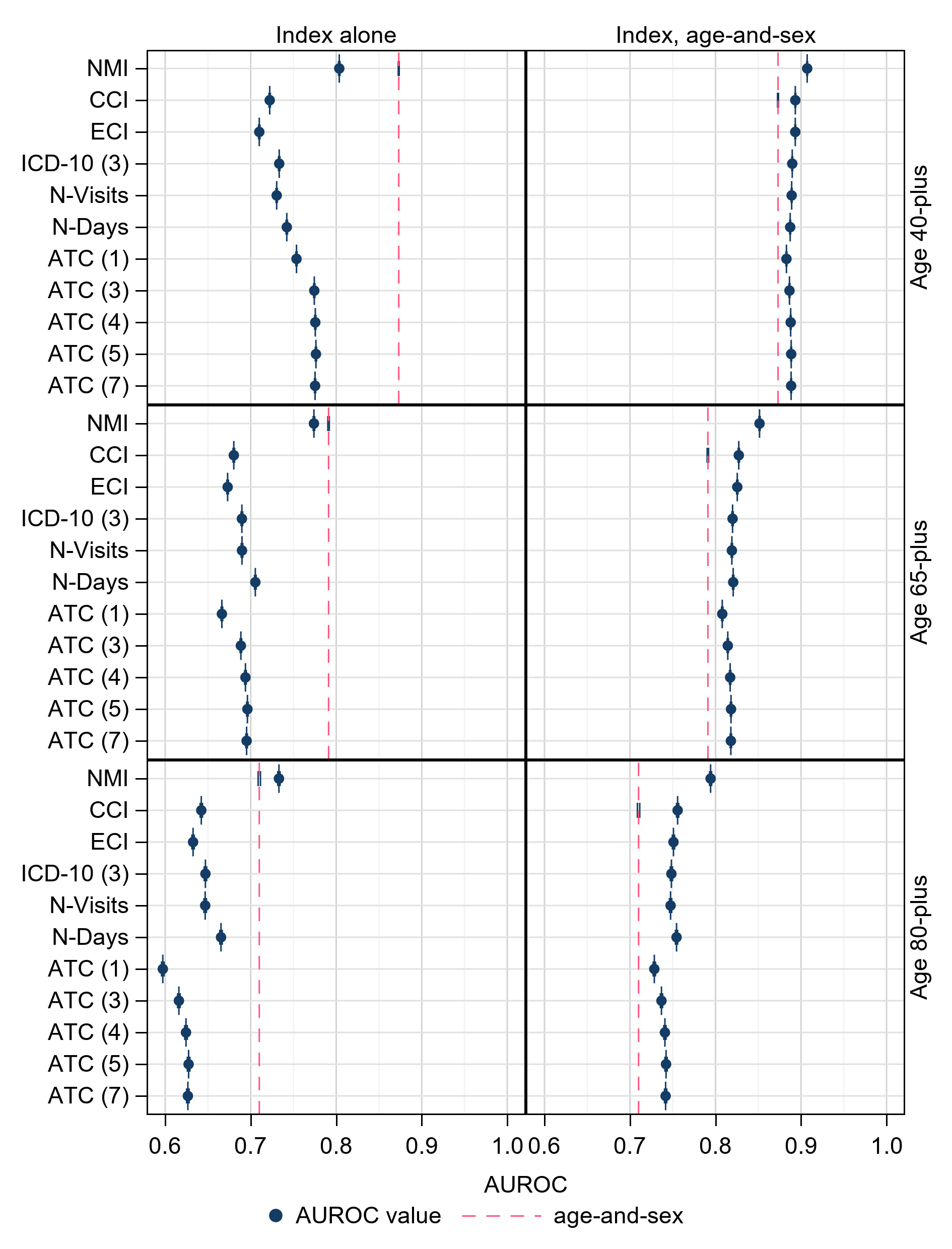** |
| --- |

**Table A5.** Numerical results corresponding to Figure 3. Area under the Receiver Operating Curve characteristics for 1- to 5-year mortality with a 1- to 5-year look-back period for diagnoses for the Nordic Multimorbidity Index (top), the Charlson Comorbidity Index (middle) and Elixhauser Comorbidity Index (bottom). Models based on (a) age-and-sex, (b) Index alone, and (c) age-and-sex *and* Index.


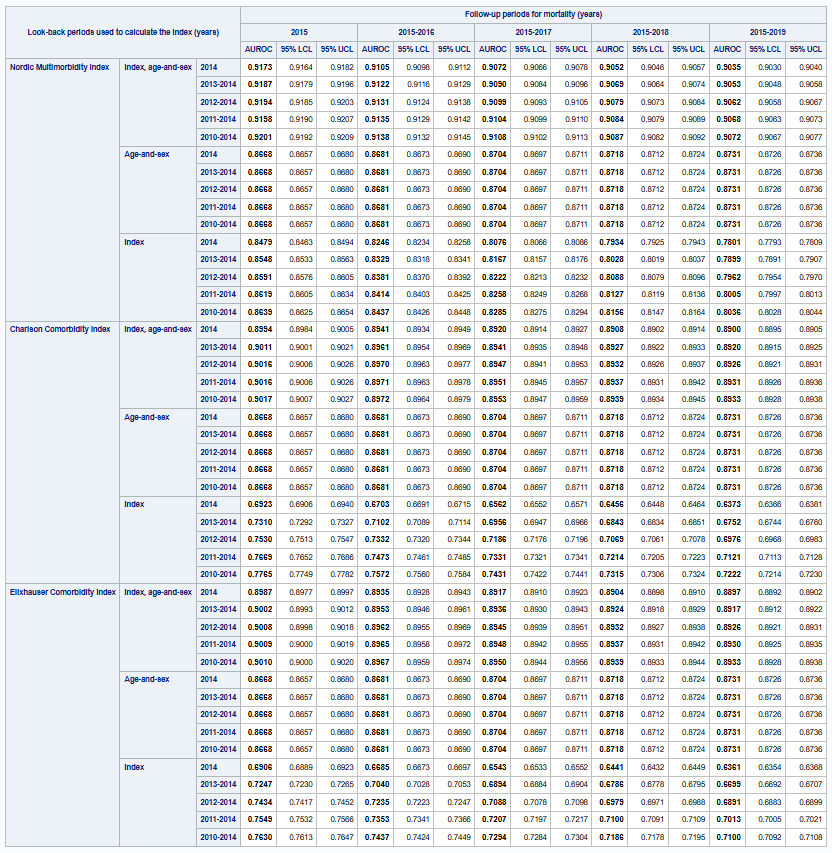


**Table A6.** Numerical results corresponding to Figure 4. Area under the Receiver Operating Curve characteristics for 1- to 5-year mortality with a 1- to 5-year look-back period for the following measures: numbers of distinct 3-character ICD-10 codes of main diagnoses (top), numbers of visits (middle) and numbers of days hospitalized for in-patient care (bottom). Models based on (a) age-and-sex, (b) measure alone, and (c) age-and-sex *and* measure.

**
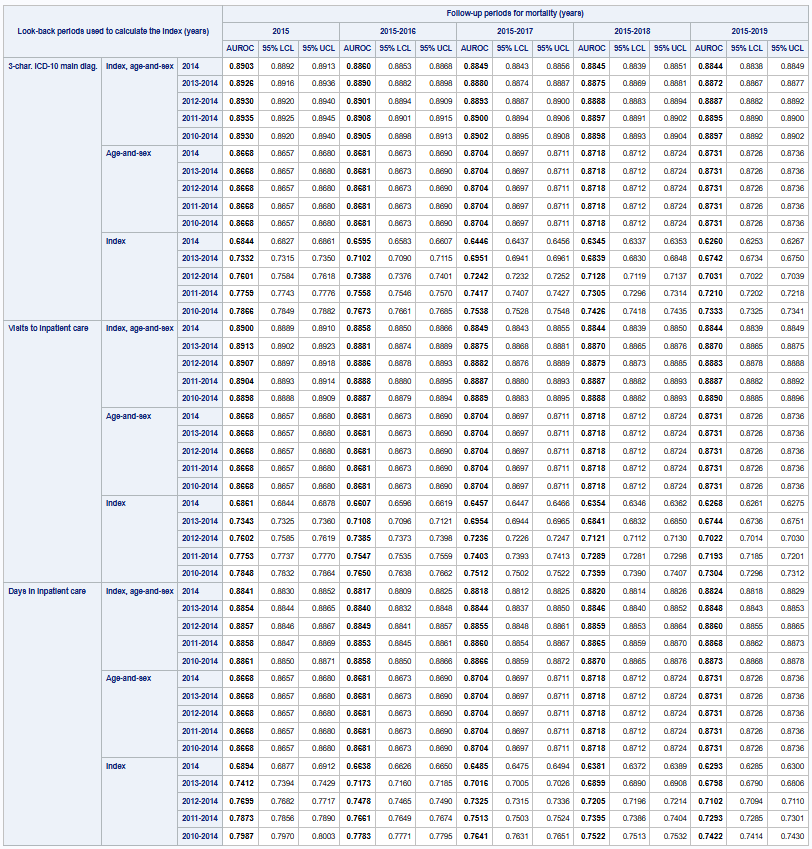
**

**Table A7.** Numerical results corresponding to Appendix Figure A1. Area under the Receiver Operating Curve characteristics for 1- to 5-year mortality with a 1-year look-back period for the following measures: numbers of distinct 1-, 3-, 4-, 5- and 7-character ATC-codes (top to bottom) on filled prescriptions from the prescribed drug register. Models based on (a) age-and-sex, (b) measure alone, and (c) age-and-sex *and* measure.

**
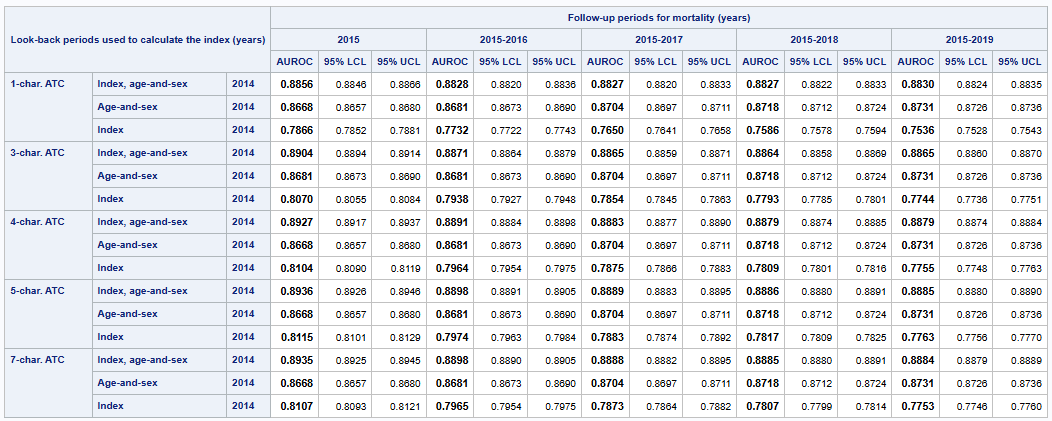
**

**Table A8.** Numerical results corresponding to Appendix Figure A2. Area under the Receiver Operating Curve characteristics for 5-year mortality in three specified age groups (years) 40-plus (top); 65-plus (middle); and 80-plus (bottom) with a 5-year look-back period for diagnoses and a 1-year look-back period for filled prescriptions (From top to bottom in each panel) for: The Nordic Multimorbidity Index, the Charlson Comorbidity Index; the Elixhauser Comorbidity Index; numbers of distinct 3-character ICD-10 codes of main diagnoses; numbers of hospitalizations; numbers of days hospitalized for in-patient care; and distinct 1-, 3-, 4-, 5- and 7-character ATC-codes. Models based on (a) age-and-sex, (b) indices and measures alone, and (c) age-and-sex *and* indices and measure, respectively.


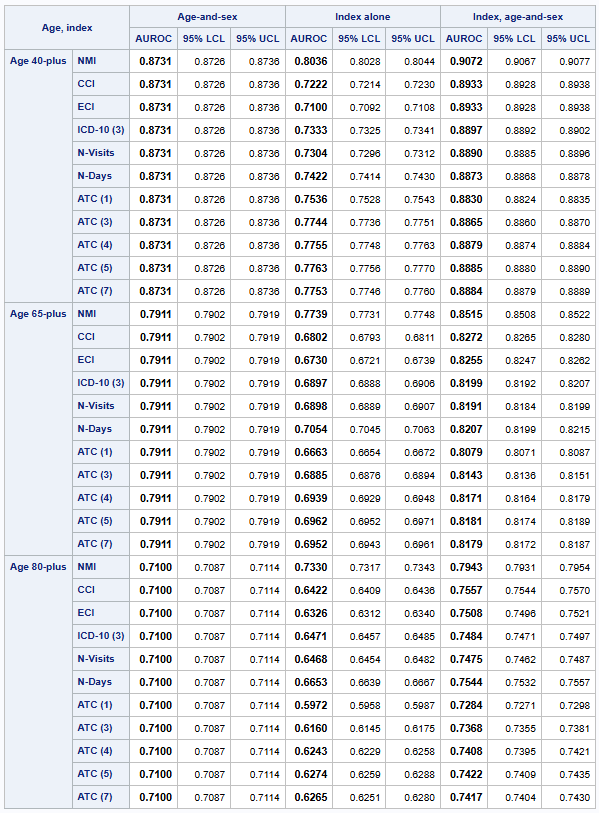

Supplement: online supplemental file 1 [file bmjopen-16-6-s001.docx]
