# Supplementary material for: Nordic Multimorbidity Index, Charlson, Elixhauser and count-based comorbidity indices for mortality risk: a comparative nationwide cohort study using national health registers in Sweden
Source: BMJ Open. 2026 Jun 22;16(6):e114635. doi: 10.1136/bmjopen-2025-114635 (PMC13288703; doi:10.1136/bmjopen-2025-114635)
Supplement: Supplementary file 2 [file bmjopen-2025-114635.reviewer_comments.pdf]

PEER REVIEW HISTORY

BMJ Open publishes all reviews undertaken for accepted manuscripts. Reviewers are asked to complete a checklist review form and are provided with free text boxes to elaborate on their assessment. These free text comments are reproduced below.

ARTICLE DETAILS

Title (Provisional)

The Nordic Multimorbidity Index, Charlson, Elixhauser and count-based comorbidity indices for mortality risk. A comparative nationwide cohort study using national health registers in Sweden Subtitle: Report from the Swedish Medical Products Agency

Authors

Zethelius, Björn; Talbäck, Mats; Ljung, Rickard

VERSION 1 - REVIEW

**Reviewer**	**1**
**Name**	**Kelson, Zoe**
**Affiliation**	**University of Exeter, Mathematics**
**Date**	**06-Jan-2026**
**COI**	**None**

The aim of the present study was to analyse Area Under the Receiver Operating Characteristic curves (AUROC) of age-and-sex alone, and in addition respectively, the Nordic Multimorbidity Index (NMI), Charlson (CCI) and Elixhauser (ECI) comorbidity indices, and explore measures based on inpatient care and filled drug prescriptions, with the outcome mortality using Swedish health registries.

Reviewer comments:

"All persons born in 1975 or before who were continuously resident in Sweden between 1st January 2010 and 31st December 2014 with a follow-up for all-cause mortality from 1st January 2015 to 31st December 2019. "

Can the authors please further comment on whether they consider the analysed cohort to be representative?

Can the authors please consider whether follow-up can be updated to more recent years?

"There were 441,254 deaths (8.9%) over the five years, 2015-2019 (Appendix 223 Tables A3). "

Can the authors please clarify whether only outcomes in the 5 year window were analysed?

What about deaths prior to 2015?

"The following registries, included in the COvid-19 VACcination register SAFEty study in Sweden (CoVacSafe-SE) platform (4), were used: Swedish National Prescribed Drug Register (PDR) (5), Swedish National Patient Register (NPR) (6, 7) and Swedish National Cause of Death Register (CDR) (8) held by the National Board of Health and Welfare and the Swedish Total Population Register (TPR) (9) and the Swedish Longitudinal Integrated Database for Health Insurance and Labour Market Studies (LISA) (10) held by Statistics Sweden"

Can the authors please comment on the completeness and coverage of the registers?

Can the authors please confirm if there was any missing data?

"AUROC were calculated with 1- to 5-year-look-back for age-and-sex, each index and explored 76 measures for 1- to 5-years-mortality" [Abstract]

and

"The discriminatory performance of the comorbidity indices was assessed using the Area Under the Receiver Operating Characteristic curve (AUROC). Models of age-and-sex alone were analysed in all follow-up settings described below and accounted for in all full model analyses, respectively. We computed the AUROC for mortality at one, two, three, four and five years of follow-up along with 95% confidence intervals (CIs)... For the five measures explored based on filled prescriptions during the year before 1st January 2015, one AUROC could be calculated for each of the five mortality outcome periods"

Can AUROCs please be statistically compared?

"Table 1. Population characteristics for the population at risk on January 1st, 2015."

Can race and ethnicity please be included in the study?

"Strengths and limitations"

Can the authors please expand on the Discussion of the study limitations, focussing on challenges and assumptions in the study design as well as scope and generalisability of the research question (rather than the study outcomes)?

Thanks for providing a copy of the STROBE checklist.

**Reviewer**	**2**
**Name**	**Blank, Robert D**
**Affiliation**	**Medical College of Wisconsin**
**Date**	**23-Feb-2026**

COI **I have leadership roles with the International Federation of Musculoskeletal Research Societies and the International Society for Clinical Densitometry. I receive editorial stipends from Elsevier and the Bone Health**

and Osteoporosis Foundation (US). I receive royalties from Wolters Kluwer. I consult for Guidepoint Advisors and the Gerson Lehman Group. I hold stock in multiple pharmaceutical, biotechnology, and medical device companies.

The goal of this MS was to compare the incremental change in predictive power when the NMI, CCI , or ECI is added to age and sex. The headline finding is that the NMI provides modest additional predictive power, with CCI and ECI both less helpful. Additional analyses vary the duration of the prediction window and the look-back period. The presentation is clear and generally well-balanced. I have some reservations about this work as outlined below:

1. The goal of the study is quite modest and the results are completely unsurprising. It is well established that age and sex are powerful predictors of mortality. With AUROC approaching 0.9, it is unsurprising that only very modest improvements are achievable using administrative data. It is similarly unsurprising that NMI was more helpful than either CCI or ECI, for the reasons laid out by the authors: development in a Scandinavian population, inclusion of more data sources, and more recent development. The same is also true regarding the greater incremental value of comorbidity data in a younger cohort.

2. All 3 of the comorbidity indices explored are count-based. The authors did not consider alternative strategies such as clustering. At the very least, omission of such approaches from consideration should be identified as a study limitation.

3. The abstract should feature the negative findings more prominently. IMO, the findings of this work are primarily negative.

VERSION 1 - AUTHOR RESPONSE

Reviewer: 1

Prof. Zoe Kelson, University of Exeter
Comments to the Author:
The aim of the present study was to analyse Area Under the Receiver Operating Characteristic curves (AUROC) of age-and-sex alone, and in addition respectively, the Nordic Multimorbidity Index (NMI), Charlson (CCI) and Elixhauser (ECI) comorbidity indices, and explore measures based on inpatient care and filled drug prescriptions, with the outcome mortality using Swedish health registries.

Reviewer comments:

Comment

"All persons born in 1975 or before who were continuously resident in Sweden between 1st January 2010 and 31st December 2014 with a follow-up for all-cause mortality from 1st January 2015 to 31st December 2019. "

-Can the authors please further comment on whether they consider the analysed cohort to be representative?

-Can the authors please consider whether follow-up can be updated to more recent years?

Response:

Thank you for this two-part question! Yes, the cohort is representative; it includes the entire Swedish population aged 40 years and above at study entry 1st January 2015. To allow for full five-year look back coverage of data from 2010 to 2014 persons also had to be continuously resident in Sweden between 1st January 2010 and 31st December 2014.

From the potential cohort of 5,631,018 individuals who had lived in Sweden at some point between 2010 and 2014, 444,167 (7.9%) who died and 176,590 (3,1%) who migrated to or from Sweden during this period were not included. See page6, rows 152 to 155 in the revised manuscript

Thus, the persons resident on 31st December 2014 that have not been resident continuously since 1st January 2010 were not included. However, if they were included, we would not have a full 5-year look-back period - which we would regard as a major disadvantage. The included registers are nationwide with high completeness and validity and are representative of the entire Swedish population.

We hesitate to consider follow-up to more recent years than 2019 due to the COVID-19 pandemic that spread across the globe early in 2020. The pandemic, defined as a threat to public health by the World Health Organisation (WHO) was declared over by the WHO as recent as in May 2023. It had consequences to all parts of life, and we cannot exclude that increased mortality during the pandemic may affect the relation between index values and mortality in analyses.

Comment

"There were 441,254 deaths (8.9%) over the five years, 2015-2019 (Appendix 223 Tables A3). "
Can the authors please clarify whether only outcomes in the 5 year window were analysed? What about deaths prior to 2015?

Response:

Thank you for his question and the possibility to clarify. Yes, deaths were only counted in the follow-up window 2015-2019. Categorization of index-data was only calculated in the 5-year look-back period 2010-2014. The follow-up of the defined cohort started on 1st January 2015 and went on for five years (See also the response above on arguments for the chosen period). Only those living from 1st January 2010 and who were still alive and residents in Sweden on 31st December 2014 were included. This was made to get reliable five-year look-back data for all persons included. Those who died during the period 2010 to 2014 are not possible to follow up for mortality from 1st January 2015. If one would like to analyse deaths during a follow up on the period 2010 to 2014 then one need similar full look back data from 2005 to 2009 (It would have included the period of the H1N1-pandemic in 2009). Taken together we wanted to use as recent data as possible however avoiding possible bias from

the COVID-19 pandemic. Please see the study population description on page 7, rows 157 to 161 in the revised manuscript.

Comment

"The following registries, included in the COvid-19 VACcination register SAFEty study in Sweden (CoVacSafe-SE) platform (4), were used: Swedish National Prescribed Drug Register (PDR) (5), Swedish National Patient Register (NPR) (6, 7) and Swedish National Cause of Death Register (CDR) (8) held by the National Board of Health and Welfare and the Swedish Total Population Register (TPR) (9) and the Swedish Longitudinal Integrated Database for Health Insurance and Labour Market Studies (LISA) (10) held by Statistics Sweden"

-Can the authors please comment on the completeness and coverage of the registers?

Response:

Thank you for the possibility to clarify on this matter. Reporting to these registers is compulsory by law in Sweden. Completeness and coverage for each of the registers is presented in respective references 5 to 10 above that were referred to in first submission. All the registers have high completeness and coverage. For example, the Cause of Death register has almost 100% completeness for Swedes who dies in Sweden and Swedes who die abroad. It was initiated in 1952. The Prescribed Drugs Register is the youngest of the registers. It was initiated in July 2005 and it has 100% coverage of filled prescriptions.

Comment

-Can the authors please confirm if there was any missing data?

Response:

We can confirm that we have received complete register data for the actual period under study, from each one of the registers included (Described above, references 5-10). Coverage of these registers is deemed very high and is presented in respective references, 5 to 10. Please see page 8, rows 185 to 189 in the revised manuscript.

Comment

"AUROC were calculated with 1- to 5-year-look-back for age-and-sex, each index and explored 76 measures for 1- to 5-years-mortality" [Abstract]
and
"The discriminatory performance of the comorbidity indices was assessed using the Area Under the Receiver Operating Characteristic curve (AUROC). Models of age-and-sex alone were analysed in all follow-up settings described below and accounted for in all full model analyses, respectively. We computed the AUROC for mortality at one, two, three, four and five years of follow-up along with 95% confidence intervals (CIs)... For the five measures explored based on filled prescriptions during the year before 1st January 2015, one AUROC could be calculated for each of the five mortality outcome periods"
Can AUROCs please be statistically compared?

Response:

Yes, AUROC can be compared. For example, AUROC for NMI can be compared to CCI. We have stated that confidence intervals were very narrow in this large dataset including >5,000,000 persons. For increased transparency we now present AUROC with 95%-confidence intervals in four new tables in the appendix in addition to and corresponding with the figures 3, 4, A1 and A2 presented in the first submission. For example, AUROC with 95% CI for NMI alone with five- year look-back and five-year follow-up was 0.8036 (0.8028 - 0.8044), Appendix Table A5, corresponding to figure 3. For further details, please see the Appendix Tables for details on the narrow confidence intervals.

Comment

"Table 1. Population characteristics for the population at risk on January 1st, 2015."
Can race and ethnicity please be included in the study?

Response:

We have no information on race nor on ethnicity, as that is not recorded in any national registers in Sweden. However, we have added information on country of birth which is available in the registers used for the study (please see below) in table 1 (Page 21, row 631 and onwards in the revised manuscript). This is descriptive data and we want to stress that this type of information is not included in the widely used Charslon comorbidity index nor in the Elixhauser index.

**Country of birth**, n (%)	Men	Women	Total
Swedish-born with two Swedish-born parents	1,655,302 (67.9)	1,645,560 (63.9)	3,300,862 (65.9)
Swedish-born with at least one Swedish-born parent	232,935 (9.6)	240,777 (9.4)	473,712 (9.5)
Swedish-born with foreign-born parents	189,917 (7.8)	277,066 (10.8)	466,983 (9.3)
1st generation immigrants born in Europe	220,633 (9.1)	264,740 (10.3)	485,373 (9.7)
1st generation immigrants born outside Europe	138,229 (5.7)	145,102 (5.6)	283,331 (5.7)

Comment

"Strengths and limitations"
Can the authors please expand on the Discussion of the study limitations, focussing on challenges and assumptions in the study design as well as scope and generalisability of the research question (rather than the study outcomes)?

Response:

Thank you for this suggestion. As a response to an editorial comment, we have added a section entitled ‘Strengths and limitations of this study’, immediately after the abstract on page 4, rows 97 to 114 in the revised manuscript.

We have further expanded this part of the discussion section, see changes in the revised manuscript on page 15, rows 410 to 426. The study design is a standard register based prospective cohort follow-up study. A limitation is the absence of data on lifestyle factors, e.g. tobacco smoking, a risk factor of CVDs and lung cancer, in the national registers. Thus, we cannot analyse the impact smoking has on mortality in this study design however we must stress that tobacco smoking is not included in the CCI or in the ECI. CVDs and cancer are however captured in the register data used.

Focussing on key challenges and assumptions: Non register based cohort studies face significant challenges including high costs, long time commitments, and high rates of participant attrition (loss to follow-up), which can threaten validity. Other major issues include confounding variables, selection bias and logistical hurdles in managing data over many years. Further, assumptions are that participants are initially free of the outcome, that accurate, stable exposure classification is present and minimal loss to follow-up, and accurate outcome measurement over time.

Key Challenges and assumptions in Cohort Studies

- Attrition rates and loss to follow-up: Participants may move, lose interest, or die, which can introduce bias if those who drop out differ from those who remain. The outcome measure mortality is captured in the Cause of Death Register used with almost complete data, nearly 100%. It has been around for the last 74 years. See reference in first submission.
- High costs and long duration: Prospective studies require extensive funding and time (years or decades), making them difficult to sustain. However, we have used health registers that have been up and running a long time before the study period chosen for the present study, see references 5 to 10 in first submission. It is compulsory by law to report to the registers. The registers are funded by the Swedish state. Thus, our study was not affected by such difficulties.
- Confounding and bias: Without randomization, groups may not be comparable. Participants might not represent the general population, affecting generalizability. Randomisation is performed in a segment of clinical trials however not in clinical medicine where therapy is allocated according to the diagnosis put and applicable treatment guidelines. In Sweden we have a publicly financed general health care and services. Thus, there is no selection bias in who get care or not. Health register data used in the present study capture the diagnoses and the filled prescriptions. The Swedish Medical Product Agency provide evidence-based treatment guidelines in Swedish for those who prescribe medicines. The data used in this study represent the total, general population above the age of 40 years a. Thus, the results are fully generalizable in Sweden. The NMI was developed in Denmark. The Nordic countries Denmark, Finland, Norway and Sweden have similar health care and registers and thus results are generalizable to a very high degree to these countries as well. The authors of the original publication on NMI called for validation. We proposed in the future perspectives part of the discussion in first submission that future research should also look at the NMI and the explorative measures performance using primary care data which we can’t at present. Further, results from the present study should be validated in other countries/settings which is beyond the scope of the present study.

- Logistical hurdles in managing data over many years. Managing large datasets, ensuring consistent data quality over time, and handling missing data are complex tasks. However, the registers used all have well established and financed management within Swedish governmental agencies. Thus, secured long-term funding is assured and possible problems in managing researcher turnover over time that may affect longitudinal studies is not at hand here. Data quality is deemed high and is regularly checked, please see references 5 to 10 in first submission for details.
- Participants must not have the outcome of interest (e.g., disease) at the start of the study. In the present study on mortality as the outcome, it is clarified that if you are dead, you cannot be included in the study if you died before study entry.
- It is often assumed that exposure status remains unchanged during the follow-up period. In this study stable exposure classification is present and there is minimal loss to follow-up, please see references number 5 to 10 in first submission on information on respective register used for defining the indices. Further, in separate analyses stratifying the amount of data used for estimation for different follow-up periods presented in the figures of first submission we presented results for five different look back periods of 1 to 5 years and five prospective periods of 1 to 5 years.
- It is assumed that participants who leave a study (attrition) are not significantly different from those who remain, meaning the loss to follow-up does not skew the results. In the present study we have used the Cause of Death Register including information on all persons for mortality calculations, independent on where they died (In Sweden or abroad). Thus, skewed results due to loss to follow-up is not expected here.
- It is assumed that the study sample is representative of the target population to ensure external validity. We included all Swedes age 40 and above in our study. This was made for reasons of congruency to the original publication of the Nordic multimorbidity index. Please, also see above the discussion in the bullet point on confounding and bias where we discuss representativeness.

<u>Scope and generalisability of the research question</u>

- The scope, i.e. the boundaries and extent of the study is outlined by the topic (mortality) and objectives (comparing the NMI, CCI and ECI, i.e. comorbidity indices for mortality prediction). We performed it in Sweden on the population aged 40 years and above to conform with the population investigated when defining the NMI in the Danish study as an objective was to compare this relatively newer NMI with the previously established CCI and ECI similarly to the Danish study. Thus, younger individuals were not included preventing the age span in the study to be too broad.
- The location, Sweden is comparable to Denmark, where the NMI was developed, when it comes to universal health care and registers.
- The time frame was chosen to not be affected by the COVID-19 pandemic which started in 2020 as we cannot exclude that analyses of relations between indices and mortality may be affected.
- Thus, we ensured study feasibility by focusing the research performed to a period that is not affected by the pandemic. This guided the data collection period.

- Generalisability of the results depends on the setting, and it is considered valid to other “free living” populations with similar access to healthcare and extrapolation to other time points in the future is reasonable. Quantitative research like this is aiming for high generalisability.
- We have presented 5-years risk estimates as in the Danish NMI-study and 1-year risk estimates as was the scope of the CCI when first published. Such shorter periods, instead of 10-years risk or more as used in many other publications, has been proposed to be more accurate and robust than for 10-year risk estimates. Further, it is more comprehensible on the patient level.
- Calibration of models, especially the NMI and the ECI was good as predictions were very close to observed mortality, as seen in figure 1 in first submission, i.e. a good calibration.

Comment

Thanks for providing a copy of the STROBE checklist.

Response:

Thank you for your peer review including constructive criticism. Possibilities to clarify and suggestions that improved our work.

Reviewer: 2

Dr. Robert D Blank, Medical College of Wisconsin
Comments to the Author:
The goal of this MS was to compare the incremental change in predictive power when the NMI, CCI , or ECI is added to age and sex. The headline finding is that the NMI provides modest additional predictive power, with CCI and ECI both less helpful. Additional analyses vary the duration of the prediction window and the look-back period. The presentation is clear and generally well-balanced. I have some reservations about this work as outlined below:

Comment

1. The goal of the study is quite modest and the results are completely unsurprising. It is well established that age and sex are powerful predictors of mortality. With AUROC approaching 0.9, it is unsurprising that only very modest improvements are achievable using administrative data. It is similarly unsurprising that NMI was more helpful than either CCI or ECI, for the reasons laid out by the authors: development in a Scandinavian population, inclusion of more data sources, and more recent development. The same is also true regarding the greater incremental value of comorbidity data in a younger cohort

Response:

Thank you for your comment. Sex and age are indeed powerful predictors of mortality. That is the reason why we first performed AUROC calculation on age and sex alone and then added respective index NMI, CCI, EXI etc. in separate models. This, approach is revealing

incremental values of each index presented in the figures enclosed in the first submission and now for increased transparency also numerically in the four tables added to the appendix. For us in the field, it is not unsurprising to find very modest improvements when AUROC for a model including age and sex alone is very high. However, the CCI is commonly used for comorbidity adjustment – maybe without reflecting on the actual added value. It is thus reasonable to question the value of using the NMI, CCI, or ECI in mortality prediction when sex and age is known, especially in the elderly groups. Therefore, we performed analyses on the whole population from age 40 years and above and the subgroups of 65 years and above and of 80 years and above, respectively, as presented in first submission. We have added a short sentence on this in the discussion part on page 12, row 338 in the revised manuscript.

Even so, the NMI was somewhat more helpful, i.e. informative for the reasons laid out by the reviewer: "*That NMI was more helpful than either CCI or ECI, for the reasons laid out by the authors: development in a Scandinavian population, inclusion of more data sources, and more recent development*" but improvement was modest. It is important to establish the value of using these complex indices. However, for comparison, we investigated "simpler" count based markers like numbers of diagnoses, numbers of inpatient care visits and days hospitalized, and numbers of filled prescriptions as presented in first submission. And they performed as well or even better. These "simpler" makers demand less granular data, are easier to calculate, and possible easier to interpret compared to more complex indexes like the CCI or ELX. We have added the wording "simple count based" on page 5, row 143 in the revised manuscript.

The reviewer points out that the incremental change in predictive power when the NMI, CCI, or ECI is added to age and sex does not add much. Still, indices are used and are frequently asked for by peer reviewers to be added for further adjustments in follow-up studies where adjustments include age, sex and major confounders of the outcome under study even though the outcome is not mortality. The CCI was developed by dr Mary Charlson in the 1980s for prediction of one-year mortality in hospitalized patients at a hospital in New York, USA. It has been used extensively and has been modified over the years to be up to date with present ICD-10 coding. It is generally considered as an important tool for confounding adjustments.

A simple PubMed search on 12 March and on 19 March 2026 gives the result in the table below. As one can see more than 16,000 publications refers to the expression "Charlson comorbidity index". Several new ones were indexed the last week. A search term, i.e. the name "Charlson" itself identify close to 20,000 publications of which Dr Mary Charlson have published 225 of them, indexed in PubMed herself. Also, probably a lot more studies have used the CCI or variants of it without citing the original paper. The table also show an interesting phenomenon that there is no stringent or unifying way of referring to Dr Chalson´s comorbidity index in pub-med indexed peer reviewed publications. In fact she didn't call it that herself when she first published in 1987 as the title was: " *A new method of classifying prognostic comorbidity in longitudinal studies: development and validation. Charlson ME, Pompei P, Ales KL, MacKenzie CR.J Chronic Dis. 1987;40(5):373-83. doi: 10.1016/0021-9681(87)90171-8. PMID: 3558716*".

It deserves however to be pointed out that her publication was an important milestone in considering comorbidity in clinical research and management. It is for example used in tailoring treatment plans for cancer treatment. Dr Charlson's publication: "*Comorbidity: From*

a Confounder in Longitudinal Clinical Research to the Main Issue in Population Management. Charlson ME, Wells MT.Psychother Psychosom. 2022;91(3):145-151. doi: 10.1159/000521952. PMID: 3519666" is discussing and reviewing comorbidity issues in more detail and from several perspectives, not to be further expressed here.

Table 1. Results from PubMed search results 12th and 19th March 2026

Search term	12th March (n)	19th March (n)
Charlson comorbidity index	16,295	16,335
Charlson comorbidity index score	8,254	8,275
Charlson index	17,136	17,177
Elixhauser comorbidity index	1,306	1,310
Nordic multimorbidity index	10	10

Further, the reviewer points out: "The same is also true regarding the greater incremental value of comorbidity data in a younger cohort". Thank you for pointing this out. It is, as observed, self-evident when you see it laid out as presented in our figure A2 in the appendix. This phenomenon is due to a lower incremental value added of an increase to a score in an older age group where co-morbid conditions are more prevalent. Fewer old individuals are without comorbidities, i.e. zero-level scores are less prevalent and scores in older age groups are on the average higher. In contrast, in younger individuals' comorbid conditions are fewer, zero-level scores are frequent, and scores are on the average lower. Comorbidity is thus a stronger marker for mortality in a younger age group. This has been added on page 12, rows 312 and 318 to 323 in the revised manuscript. It may, at a first glance, seem counterintuitive that incremental value is lower in the elderly without reflecting on the actual added value of an increment of one in a score in relation to its range and average value.

We suggest adding your headline above "*the NMI provides modest additional predictive power, with CCI and ECI both less helpful*" with a slight variation to the text in the conclusion on page 16, row 445 as well as in the conclusion of the abstract, page 3, rows 89 to 90.

<u>Comment</u>

2. All 3 of the comorbidity indices explored are count-based. The authors did not consider alternative strategies such as clustering. At the very least, omission of such approaches from consideration should be identified as a study limitation.

<u>*Response:*</u>
Yes, the indices NMI, CCI and ECI analysed are all count based and weighted scores and a higher score indicates a higher predicted risk of mortality. The simpler scores that we explored were all count-based. The aim of the present study was to compare the established scores and their incremental values when added to the powerful predictors of death, age and sex and further explore simple scores that do not need complex calculations, as presented in the first submission. The aim was not to develop a new cluster-based score thus it is not considered as a limitation to the present study. However, pre-existent chronic diseases may be grouped into different categories like individuals with malignant cancers, cardiovascular diseases, glucometabolic disease, COPD, nephro- and hepatic diseases, dementia, psychiatric disease or a low-multimorbidity group and so on. Such clusters may distinguish

individuals with advanced, complex, or late-stage disease from those having earlier-stage disease. One must bear in mind that registers capture type of disease but not the degree of disease. However, both the NMI and the CCI use weights derived to reflect degree of severity of disease burden. The NMI-weights were shown in the appendix in the first submission. The CCI assigned weights of 1, 2, 3, or 6 are based on mortality risk and are well known. Thus, these indices weigh degree of disease burden in relation to mortality risk and are directly optimized for mortality prediction. Score-based algorithms generally outperform clustering for mortality prediction in register-based data because they directly model risk, while clustering is better for discovering disease patterns rather than predicting outcomes.

We performed an exploratory analysis of numbers of filled prescriptions the last year, i.e. a count-based measure. We used Anatomical Therapeutic Chemical (ATC) Classification System codes available in the prescribed drugs register. ATC is an established acronym for the codes defined by the World health Organisation [Anatomical Therapeutic Chemical (ATC) Classification] and frequently used in pharmaco-epidemiological research. It performed nearly as good as the indices that are needing more complex calculations. In some way there is an analysis of clustering performed as we analysed ATC-codes of different numbers of characters from 1 to 7 where the former is a cluster of the ATC-codes with higher numbers of characters within the category, i.e. the same initial letter. Please, see the WHO reference (12) in first submission for further details on ATC- codes.

If the reviewer means a cluster-based score on pre-existent chronic diseases grouped into different categories such an approach is possible using ATC-code information. That is a scope for another future work and was, as stated not the aim of the present article. We thus prefer to point out that cluster-based score analyses using ATC-code information should be explored further under the subheading future perspectives in the discussion section, please see page 15, rows 436 to 438 in the revised manuscript.

Comment

3. The abstract should feature the negative findings more prominently. IMO, the findings of this work are primarily negative.

Response:

Thank you for this comment. We have taken the opportunity to milder the abstract somewhat in the revised version as the magnitude of findings are modest. However, the wording “negative” is somewhat puzzling to us as researchers with experience of regulatory work where the term “negative” is used for a failed clinical study like an adequately powered RCT that does not show a statistically significant difference of clinically meaningful magnitude and not for a comparative or exploratory epidemiological study. The latter can identify a magnitude of a relationship with a certain degree of confidence, often expressed as a 95%-confidence interval. As seen in the figures in first submission the 95%-confidence intervals are very narrow due to the very large sample size. However, we provide for full transparency in the revised Appendix four tables including AUROC and 95%-CIs corresponding to results presented in figures 3, 4, A1 and A2 in the first submission.

Finally, we want to thank both reviewers for their time and effort performing their peer review including constructive criticism which made it possible to clarify the study results further. We

understand that the reviewers' comments, our responses, and the previous version of our article will be published as supplementary information alongside the final version of our article if the decision of the editor is to accept it for publication.

VERSION 2 - REVIEW

**Reviewer**	**1**
**Name**	**Kelson, Zoe**
**Affiliation**	**University of Exeter, Mathematics**
**Date**	**27-Mar-2026**
**COI**	

Many thanks to the authors for responding to each reviewer comment in turn, satisfactorily providing clarification, and revising the article where required.

**Reviewer**	**2**
**Name**	**Blank, Robert D**
**Affiliation**	**Medical College of Wisconsin**
**Date**	**05-Apr-2026**
**COI**	

Thanks to the authors for revising the MS. It is notably improved and more balanced, addressing the key issues raised in review of the original MS.
